# Significance of mandibular molar replacement with a dental implant: a theoretical study with nonlinear finite element analysis

**DOI:** 10.1186/s40729-018-0117-7

**Published:** 2018-02-27

**Authors:** Masazumi Yoshitani, Yoshiyuki Takayama, Atsuro Yokoyama

**Affiliations:** 1 Division of Oral Functional Science, Department of Oral Functional Prosthodontics, Graduate School of Dental Medicine, Hokkaido University, Kita-13, Nishi-7, Kita-ku, Sapporo, 060-8648 Japan; 2 Removable Prosthodontics, Hokkaido University Hospital, Hokkaido University, Kita-14, Nishi-5, Kita-Ku, Sapporo, 060-8648 Japan

**Keywords:** Dental implant, SDA, Occlusal force distribution, Finite element analysis

## Abstract

**Background:**

Dental implants are frequently applied to unilateral defects in the mandible. However, implant placement in the molar region of the mandible can be difficult due to anatomical structure. The aim of this study was to evaluate the distribution of occlusal force in a mandibular shortened dental arch (SDA) with implants.

**Methods:**

Three-dimensional finite element (FE) models of the mandible with varying numbers of teeth and implants were constructed. Models Im6 and Im67 contained one and two implants in the defect of the left molar region, respectively. Models Im456 and Im4567 contained three and four implants in the defect of the left premolar and molar regions, respectively. Model MT67 contained a defect in the molar region with no implant placed. Model MT7 represented natural dentition without a left second molar, as a control. Modification of the condition of occlusal contacts assuming the intercuspal position was performed before analysis under load 400 N; therefore, the load condition as total force on the occlusal surface was 400 N. FE analyses were subsequently performed under load conditions of loads 100, 200, and 800 N. The distribution of reaction forces on the occlusal surface and the mandibular condyle was investigated.

**Results:**

Force distribution in models Im67 and Im4567 appeared to be symmetrical under all load conditions. Occlusal force distribution in models Im6 and Im456 was similar to that in model MT7. However, the occlusal force at the second premolars on the defect side in those models was larger under loads 100 and 200 N. Conversely, the occlusal force on the first molars was much larger than that in model MT7 under load 800 N.

**Conclusions:**

Within the limitations of this theoretical study, we demonstrated that restoration with the same number of implants as missing teeth will show almost symmetric occlusal force distribution, and it will produce less biomechanically stress for a unilateral defect of the mandible. However, if restoration of a missing second molar with an implant is impossible or difficult, then an SDA with implants may also be acceptable except for individuals with severe bruxism.

## Background

Dental implant treatment has been frequently applied in dental practice as the most important prosthodontic procedure with long-term predictability to restore oral function, maintain occlusion, and improve the quality of life (QoL) of a patient [1]. Clinically, dental implants are mainly applied to correct mandibular distally extended edentulism [2]. However, implant placement in the molar region of a mandible occasionally has some anatomical difficulties, such as lingual concavity of a mandible, small distance to the mandibular canal, insufficient space between the alveolar ridge and opposing teeth, and lack of keratinized mucosa. Especially, lingual concavities in an edentulous mandible appear to be related to risk of perforation in the lingual cortical bone during dental implant insertion, which may lead to hemorrhages or infections in the parapharyngeal space [3, 4]. Lingual concavities have a prevalence of 68% in the molar region and occur at a significantly higher rate in the second molar (90%) than in the first molar (56%) [5]. To avoid these risks, implants may not always be suitable to repair second molar defects.

Dental implant treatment is also associated with higher initial costs in general [6, 7]. Therefore, a cost-effective treatment is desired. Removable partial dentures with a single posterior implant could be a possible treatment option in the case of inappropriate implant placement in the second molar region. However, the least amount of Oral Health Impact Profile improvement was observed in patients with removable partial dentures compared with patients with implant-supported fixed prostheses [8]. Thus, patients who desire a fixed prosthesis may not be satisfied with a removable overdenture.

The shortened dental arch (SDA) is known as an acceptable concept in natural dentition for its lower cost than restoration of missing teeth. Kayser et al. found no significant differences between subjects with SDA of three to five occlusal units (OUs) and those with complete dental arches with regard to masticatory ability, signs, and symptoms of temporomandibular disorders, migration of remaining teeth, periodontal support, and oral comfort [9–11]. Therefore, according to this concept, second molar defects may not need to be replaced. Fueki et al. identified that only 3% of subjects missing just the second molar(s) sought prosthetic treatment compared with 58% of subjects missing first and second molars [12]. Baba et al. investigated the relationship between patterns of missing OUs and oral health-related QoL in subjects with SDA and reported that a significant difference was observed between groups with and without first molar occlusal contact [13]. Although these studies have examined SDA in natural dentition, there are few studies on SDA including dental implants.

From the viewpoint of occlusal force distribution, when a second molar defect remains without prosthesis, the force might concentrate in the implant, residual teeth, or temporomandibular joints (TMJs). Therefore, the aim of this study was to investigate occlusal force distribution in SDA in the mandible with/without an implant using a three-dimensional (3D) finite element model (FEM).

## Methods

### Finite element models

The 3D FEMs were constructed based on those reported by Kasai et al. [14], Kayumi et al. [15] and consisted of a mandible, natural teeth with the periodontal ligament (PDL), and titanium implant(s) with superstructures in the left premolar and molar regions.

The surface of the mandible was generated using measurements of a commercially available model (QS7, SOMSO) of the dentate mandible with a 3D laser scanner (LPX-250, Roland DG). Appropriate thickness of the cortical bone was determined according to the anatomical findings [16, 17] and given with computer-aided design software (Rhinoceros, AppliCraft). The mass/volume and shape of the mandible were assumed to be 2 and B, respectively, according to the classification of Lekholm and Zarb [18]. The dimensions of the natural teeth and PDL were based on previous literature [19, 20]. The PDL had approximately the same surface area as the anatomical value, with uniform thickness of 0.25 mm at all sites [20]. The diameter and the length of the implants measured 3.75 and 10 mm, respectively. All materials were assumed to be linear and isotropic except for the PDL, which had biphasic properties as previously described [21–24]. The properties of other materials were based on previous studies [25–29] (Table 1).

**Table 1:** Material properties

Material	Modulus of elasticity (MPa)	Poisson ratio	References
Cortical bone	140,000	0.3	Kunavisarut et al. [26]
Cancellous bone	7900	0.3	Kunavisarut et al. [26]
Enamel	80,000	0.3	Korioth and Hannan [24]
Dentin	17,600	0.25	Korioth and Hannan [24]
Implant (titan)	117,000	0.32	Kunavisarut et al. [26]
Superstructure (gold alloy)	94,000	0.3	Van Zyl et al. [25], Kitamura et al. [28], Kobayashi et al. [27]
PDL phase1	0.33	0.3	Kim et al. [23], Misch [22], Perfitt et al. [20], Miura et al. [21]
PDL phase 2	16	0.45	Kim et al. [23], Misch [22], Perfitt et al. [20], Miura et al. [21]

**Fig. 1:**
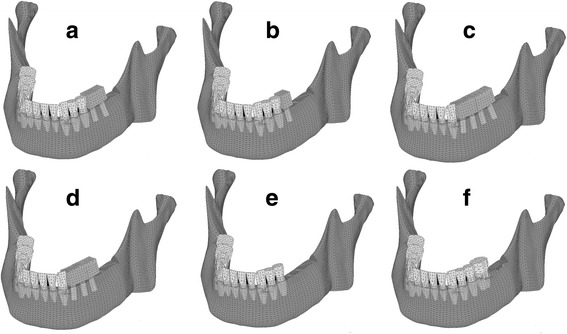
Three-dimensional finite element model. The tooth roots and implant bodies are displayed with permeability. **a** Im67, **b** Im6, **c** Im4567, **d** Im456, **e** MT67, and **f** MT7

The occlusal surfaces of the implants and the teeth were simplified and flattened in agreement with Monson’s sphere (10 cm diameter) and included two condyle points and the incisal point. Six models with varying numbers of teeth and implants were constructed. Model Im67 contained two implants placed in the left molar region (Fig. 1a). Model Im6 contained one implant placed in the left molar region (Fig. 1b). Model Im4567 contained four implants placed in the left premolar and molar regions (Fig. 1c). Model Im456 contained three implants placed in the left premolar and molar regions (Fig. 1d). Model MT67 contained no implant placed in the molar defect (Fig. 1e). Model MT7 represented natural dentition without the second molar as a control (Fig. 1f).

### Validation of models

**Fig. 2:**
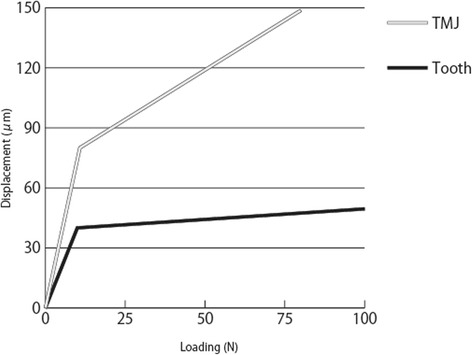
Load displacement curves of springs

Nonlinear characteristics according to the load displacement curve of teeth [21–24] and cartilage [30] were given to the springs for the opposing teeth and TMJs, respectively (Fig. 2). The nonlinear elasticity of the springs on the teeth and implants simulated displaceability of opposing natural teeth at compression and separation of the occlusal surface from opposing teeth at tension.

**Fig. 3:**
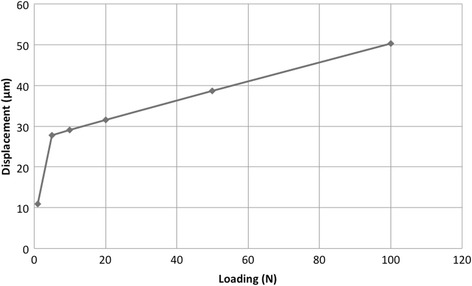
Load displacement curves of natural teeth in FE model

The PDLs of natural teeth and the springs corresponding to opposing teeth demonstrated a two-stage displaceability corresponding to the measurement of the load displacement curve of real teeth [21, 22] (Fig. 3).

**Fig. 4:**
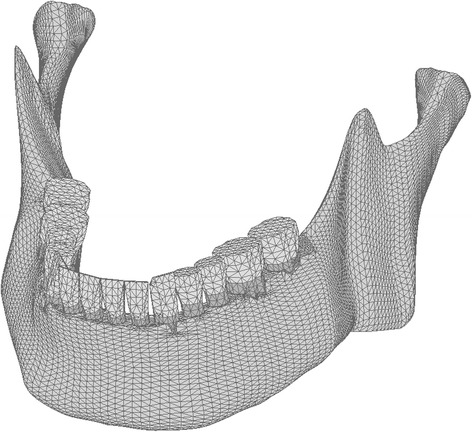
Three-dimensional finite element model with natural teeth and no defect

**Fig. 5:**
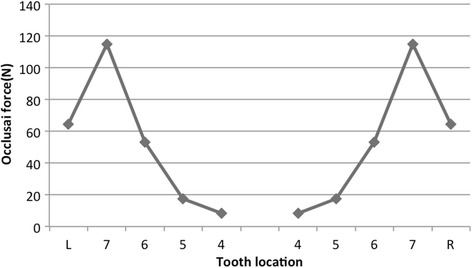
Distribution of occlusal force in the natural teeth model displayed in Fig.4

Kumagai et al. [31] investigated occlusal force distribution in human using the Dental Prescale System. They described that the distribution of the occlusal force was greatest at molar region followed by the premolar and anterior region. Our theoretical model was based on Kasai [14] and Kayumi’s [15] reports. The theoretical model with natural teeth and no defect in Kayumi’s report and the occlusal force distribution in this model under load 400 N described later are shown in Figs. 4 and 5, respectively. This distribution was similar to the measurement in human body by Kumagai et al. [31].

### Boundary conditions of models

**Fig. 6:**
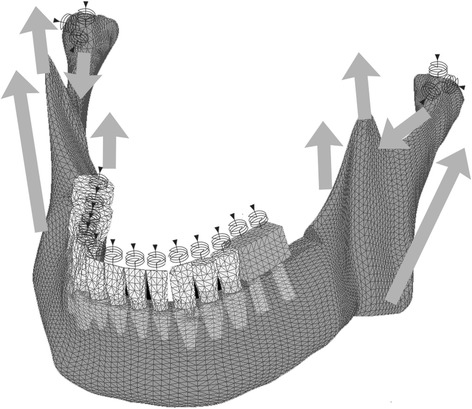
Springs for opposing teeth and TMJs and load directions. Arrows indicate loads, arrowheads indicate restricted nods, and spiral lines indicate springs

The boundary conditions are shown in Fig. 1a–f. To simplify the FEM, TMJs and maxillary teeth were replaced with appropriate springs (Fig. 6). The springs for the maxillary teeth, except for the anterior teeth, were directed perpendicular to the occlusal plane, which was defined using Monson’s sphere. The apical end of each spring was restrained in all directions. The other end of the spring was attached to the node corresponding to the occlusal central pit on a mandibular tooth, which allowed displacement perpendicular to the occlusal plane. The springs for TMJs linked an external restricted node to the top of the mandibular condyle [14, 29].

### Loading conditions

The loading conditions simulated intercuspal clenching. With the assumption that occlusal force was generated by the contractile force of four bilateral masticatory muscles, masseter, temporalis, and medial and lateral pterygoids, loading points and directions of the loads were generated based on previous literature [19, 25] (Fig. 6). The summation of the reaction force at the occlusal surfaces of the teeth and superstructures was used as a standard for the load amount. For example, the load condition that resulted in a total reaction force of 400 N in the preliminary FEM with natural dentition was designated as “load 400 N.”

### Analysis

Analysis was performed according to the report by Kayumi et al. [15]. In linear finite element analysis (FEA), all teeth maintain perfect contact with antagonists with no stress on occlusal surfaces before loading. However, there must be some occlusal force on the occlusal surface when a mandible is in the intercuspal position. Since the displaceabilities of osseointegrated implants, natural teeth with PDLs, and TMJs are quite different from one another, the results of linear FEA under some occlusal loads should not correspond to real biomechanical conditions.

**Fig. 7:**
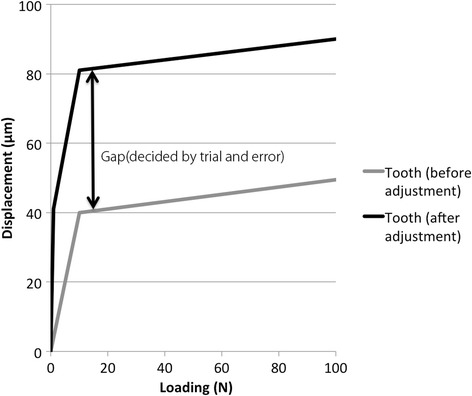
Initializing models altering the load displacement curves of springs

To verify the similarity of the FE models to the real stomatognathic system, “initializing” of models, i.e., modification of the condition of occlusal contact assuming the intercuspal position, was performed before analysis. This procedure was performed by modifying occlusal contacts on the implants so that the distribution of occlusal force was symmetrical under load 400 N [15]. The occlusal contacts were modified by altering the load displacement curves of the springs on the implants. The load displacement curve was shifted so that the spring provided little resistance to compressive forces until the gap closed, i.e., occlusal surface came in contact with antagonists (Fig. 7). The size of the gap was determined by trial and error, such that the occlusal force, namely, the reaction force of the springs on the occlusal surface, was distributed with approximate symmetry [14, 15] in models Im6, Im67, Im456, and Im4567. This size was determined so that the amount of reaction force on the most posterior teeth on both sides became as equal as possible. After initializing was completed, symmetric distribution of the reaction force on the teeth and the superstructures was confirmed in each model. Thereafter, the FEA was performed under the load conditions of loads 100, 200, and 800 N using the software package MSC.Marc2010 (MSC Software). The distribution of the reaction forces on the occlusal surface and the mandibular condyle, which were regarded as the occlusal force and the load on the TMJ, respectively, was investigated.

## Results

**Fig. 8:**
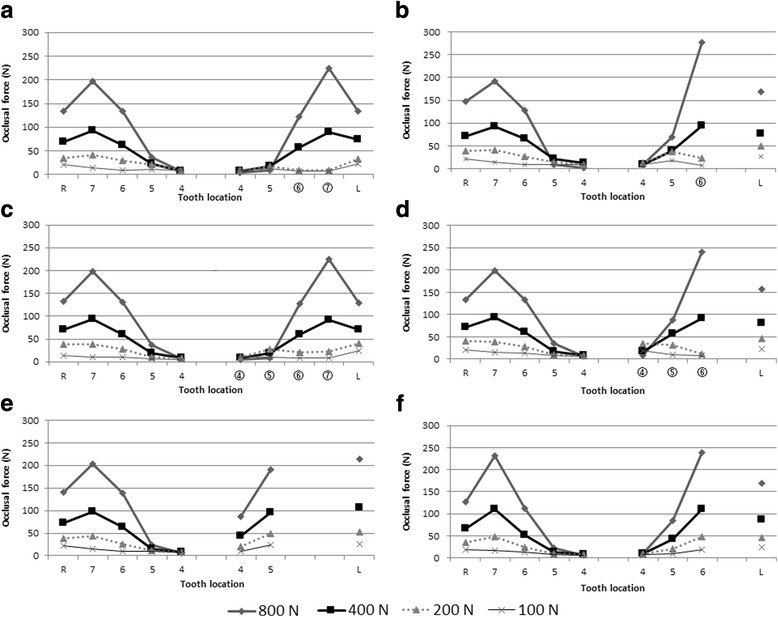
Distribution of occlusal force in models. **a** Im67, **b** Im6, **c** Im4567, **d** Im456, **e** MT67, and **f** MT7. *R* right TMJ, *L* left TMJ, *4* first premolar, *5* second premolar, *6* first molar, and *7* second molar. Numbers within circles indicate implant superstructure

The distributions of occlusal force are shown in Fig. 8. In model MT67 (Fig. 8e), the occlusal force at the first premolar on the defect side was 10.0–86.5 N, which was 1.2–15.0-fold larger than that on the natural dentition side. The occlusal force at the second premolar on the defect side was 24.6–190.1 N, which was 2.6–8.3-fold larger than on the natural dentition side. The occlusal force at the TMJ on the defect side was 26.8–214.6 N, which was 1.2–1.5-fold larger than that on the natural dentition side. The occlusal force was concentrated at the second premolar on the defect side and approximately equivalent to that at the second molar on the natural dentition side.

In model MT7 (Fig. 8f), the occlusal force at the first premolar on the defect side was almost equivalent to that on the natural dentition side. The occlusal forces at the second premolar under loads 100, 200, 400, and 800 N were 8.6, 21.0, 43.2, and 84.5 N, respectively. These forces were 1.1–3.8-fold larger on the defect side than that on the natural dentition side. Additionally, the occlusal forces at the first molar under loads 100, 200, 400, and 800 N were 18.5, 47.2, 110.7, and 239.0 N, respectively. These forces were 1.5–2.1-fold larger on the defect side than that on the natural dentition side. The occlusal force at the TMJ on the defect side was 24.9–169.1 N, which was 1.1–1.3-fold larger than that on the natural dentition side. The occlusal force was concentrated at the first molar on the defect side and approximately equivalent to that at the second molar on the natural dentition side.

In model Im67 (Fig. 8a), the occlusal force at the premolars and the TMJ on the defect side was smaller than that in model MT67 (shown in Fig. 8e). The occlusal force at the second premolar and TMJ in model Im67 was 9.7–18.3 and 23.7–134.3 N, respectively. Compared with model MT67, the occlusal force at the second premolar and left TMJ in model Im67 was reduced by 0.005–0.5-fold and 0.6–0.9-fold, respectively. Under loads 100 and 200 N, the occlusal force at the implants was slightly smaller than that on the natural dentition side. Under load 800 N, the occlusal force at the first and second molars was 122.7 and 224.9 N, respectively. The occlusal force at the implants was slightly larger than that at natural teeth on the contralateral side. However, occlusal force distribution in model Im67 was considered to be almost symmetrical.

In model Im6 (Fig. 8b), the occlusal force at the second premolar under loads 100, 200, 400, and 800 N were 18.3, 37.0, 38.8, and 70.2 N, respectively. The occlusal force was larger than that in model Im67 (shown in Fig. 8a), while it was approximately equivalent to that in model MT7 (shown in Fig. 8f). Under loads 100 and 200 N, the occlusal force at the second premolar on the defect side was larger than that at the implant. However, it was smaller than that at the right second molar (most posterior tooth on the natural dentition side). Under load 800 N, the occlusal force at the implant was 276.9 N, which was 1.4-fold larger than that at the most posterior tooth on the natural dentition side. Compared with model MT67, the occlusal force concentration at the second premolar was reduced but remained 2.0–8.3-fold larger than that on the contralateral side.

Comparing model Im6 with model MT7, the occlusal force at the second premolar on the defect side was 1.9-fold larger under load 100 N and 2.8-fold larger under load 200 N. Under load 800 N, the occlusal force at the implant was much larger than that at the first molars on both sides in model MT7. However, occlusal force distribution in model Im6 was similar to that in model MT7.

In model Im4567 (Fig. 8c), under loads 100 and 200 N, the occlusal force at the premolars on the defect side was slightly larger than that on the natural dentition side. Conversely, the occlusal force at the molars on the defect side was slightly smaller than that on the natural dentition side. Under load 800 N, the occlusal force at the second premolar on the defect side was reduced and that at the second molar on the defect side was increased. However, occlusal force distribution in model Im4567 was considered to be almost symmetrical. The occlusal force at the TMJ was symmetrical under every load condition.

In model Im456 (Fig. 8d), under loads 100 and 200 N, the occlusal force at the premolars on the defect side was larger than that in model Im4567 (shown in Fig. 8c). The occlusal force was also larger than that in model MT7 (shown in Fig. 6f). However, the occlusal force at the second premolar under load 200 N was 34.5 N, which was slightly smaller than the occlusal force at the second molar on the natural dentition side. Under load 800 N, the occlusal force at the first molar was increased to 240 N. However, it was almost equivalent to that in model MT7 (shown in Fig. 6f). The occlusal force at the TMJ was slightly increased compared with that in model Im4567 (shown in Fig. 8c).

## Discussion

### FEM

FEA is useful for mechanical simulations of a living body and has been used in implant dentistry research under careful consideration of the analysis conditions [32, 33]. Although some reports have demonstrated that bone density varies according to bone type and location, the material properties of the mandible were homogenous and isotropic in this study. However, the effect of this difference was considered to be negligible under the confirmation of the displacement of teeth and implants because of its far larger elastic modulus and far smaller strain than those of soft tissues, such as TMJs and PDL. Since the purpose of the present study was to examine the distribution of occlusal forces on the occlusal surface, occlusal forces should be mainly affected by the displaceability of TMJs and teeth, not by that of osseointegrated implants.

The FEMs in this study were based on those reported by Kasai et al. [14] and Kayumi et al. [15]. The displaceability of TMJs was regarded as that of the cartilage [30] because it has a far smaller elastic modulus than that of the TMJ disc [34, 35]. Therefore, the elastic modulus of the springs corresponding to TMJs was determined based on the thicknesses of the TMJ disc [36] and articular cartilage [35], the stress-strain curve of the intervertebral discs [30], and the displacement of the condyle [37, 38] in intercuspal clenching by indirect measurement. Although the material properties of the human body vary on an individual basis, the models used in this study were therefore considered to be appropriate to investigate the distribution of occlusal forces on the teeth, implants, and TMJs.

### Loading condition

Based on previous literature [31, 39], occlusal loading of 200 N was considered to correspond with hard clenching. However, a previous study indicated that the maximum biting force (400 N) was better for occlusal adjustment with intercuspal clenching. Therefore, this study was performed with the assumption that the maximum functional force was 400 N. Occlusal loading of 100 N was considered to correspond with light clenching while loading of 200 N was considered to correspond with middle clenching. Calculations were also performed under a load of 800 N, which was assumed to be the maximum nonfunctional occlusal force, such as that exerted in nocturnal bruxism. Because of the difficulty of controlling nocturnal bruxism, this value was considered to be sufficient to include as the condition under maximum force [40]. However, Hattori et al. [41] described that neuromuscular regulatory systems control maximum clenching strength under various occlusal conditions. Therefore, the large force used in this study may not occur clinically in the SDA except in the case of nocturnal bruxism.

### Occlusal force distribution in dentition

Occlusal adjustment is usually performed to obtain symmetrical occlusal force distribution in natural dentition. However, occlusal force distribution among natural teeth and implants depends on occlusal force because of the difference of displaceability between a natural tooth and an implant [14, 15]. Therefore, we evaluated the result of the analysis from viewpoints of symmetry of occlusal force distribution and existence of harmful load on implants, residual teeth, and TMJs.

In this study, occlusal force distribution under load 400 N was adjusted to maintain symmetry. In models Im67 and Im4567, occlusal load on premolars did not show large asymmetry even in lower and higher loading conditions. Thus, restoration with implants of the same number of missing teeth was recommended for unilateral defect(s) of the mandibular premolar and molar regions. In models Im6 and Im456, occlusal force distribution was altered more than in models Im67 and Im4567. However, occlusal force distribution in models Im6 and Im456 was similar to that in model MT7. Since most patients missing only second molars do not always receive replacements, as described above [12], restoring mandibular distally extended edentulism with a second molar defect might be acceptable, similar to receiving no prosthetic treatment for a second molar defect in natural dentition. However, a much larger occlusal force on the superstructure of the first molar was observed compared with the first molar on the natural dentition side of model Im6, the first molar on the natural dentition side of model Im456, and the first molar on the defect side of model MT7 under load 800 N. These finding might indicate a risk of damage to implants and surrounding bone tissue.

### Effect on TMJs

In this study, force distribution on the left TMJ (defect side) was similar to that on the right TMJ (natural dentition side) under not only load 100 N but also other loading conditions. Hattori et al. [41] did not find evidence that the SDA causes overloading of the joints or the teeth, which suggests that neuromuscular regulatory systems control maximum clenching strength under various occlusal conditions. Reissmann et al. [42] also demonstrated no significant difference between SDA patients and removable dental prosthesis patients. Therefore, apprehension concerning overloading of TMJs in an SDA with implants is considered to be negligible.

### Limitations of this study

It should be noted that our results were obtained under conditions of vertical loading by bilaterally balanced muscle activity with tight intercuspation in the correct mandibular position because the horizontal displacement of the premolars and molars was restrained. The actual distribution of occlusal forces may differ due to individual differences in the material properties of the soft tissue. Additionally, the lateral load, which may occur in lateral movement of the mandible during mastication, was not considered. Furthermore, this study investigated only the biomechanical aspect of the SDA using implants. The function and QoL of real patients with SDA should be investigated in future studies. Moreover, it is unclear whether the occlusal force of each tooth and implant observed in this study is harmful in human. Further investigation should include analysis of bone strain.

## Conclusions

Within the limitations of this theoretical study, we demonstrated that restoration with the same number of implants as missing teeth shows almost symmetric occlusal force distribution, and it produced less biomechanically stress for a unilateral defect of the mandible. However, if restoration of a missing second molar with an implant is impossible or difficult, then an SDA with implants may also be acceptable except for individuals with severe bruxism.
